# Cross-Species Functional Genomic Analysis Identifies Resistance Genes of the Histone Deacetylase Inhibitor Valproic Acid

**DOI:** 10.1371/journal.pone.0048992

**Published:** 2012-11-14

**Authors:** Rakel Brendsdal Forthun, Tanima SenGupta, Hanne Kim Skjeldam, Jessica Margareta Lindvall, Emmet McCormack, Bjørn Tore Gjertsen, Hilde Nilsen

**Affiliations:** 1 Institute of Medicine, Hematology Section, University of Bergen, Bergen, Norway; 2 The Biotechnology Centre, University of Oslo, Oslo, Norway; 3 Hematology Section, Department of Medicine, Haukeland University Hospital, Bergen, Norway; Institut national de la santé et de la recherche médicale (INSERM), France

## Abstract

The mechanisms of successful epigenetic reprogramming in cancer are not well characterized as they involve coordinated removal of repressive marks and deposition of activating marks by a large number of histone and DNA modification enzymes. Here, we have used a cross-species functional genomic approach to identify conserved genetic interactions to improve therapeutic effect of the histone deacetylase inhibitor (HDACi) valproic acid, which increases survival in more than 20% of patients with advanced acute myeloid leukemia (AML). Using a bidirectional synthetic lethality screen revealing genes that increased or decreased VPA sensitivity in *C. elegans*, we identified novel conserved sensitizers and synthetic lethal interactors of VPA. One sensitizer identified as a conserved determinant of therapeutic success of HDACi was *UTX (KDM6A)*, which demonstrates a functional relationship between protein acetylation and lysine-specific methylation. The synthetic lethal screen identified resistance programs that compensated for the HDACi-induced global hyper-acetylation, and confirmed MAPKAPK2, HSP90AA1, HSP90AB1 and ACTB as conserved hubs in a resistance program for HDACi that are drugable in human AML cell lines. Hence, these resistance hubs represent promising novel targets for refinement of combinatorial epigenetic anti-cancer therapy.

## Introduction

Epigenetic changes in cancer involve cooperation of multiple processes including covalent modification of histones, where histone acetylation and methylation are among the modifications shown to contribute to epigenetic reprogramming in cancer [1], [2], [3]. Histone deacetylase inhibitors (HDACi) have antitumor potential [4] and represent important therapeutic supplements in acute myeloid leukemia (AML) [5], [6] where the need for effective low toxic therapy in an elderly patient population is critical [7]. Although current HDACi are criticized for being too unspecific, they have properties recommending them as therapeutic drugs, such as low general toxicity and promising effects at low doses. Moreover, many HDACis show synergy with standard chemotherapy [8]. The histone deacetylase class I and II inhibitor valproic acid (VPA) is an example of a well-tolerated anticonvulsant with a safety profile that allows long-term use in children [9]. VPA affects cell growth, differentiation and apoptosis [10], [11] and is well tolerated in combination with chemotherapeutics and targeted therapy [12], [13], [14], [15], [16]. Myelodysplastic syndromes (MDS) and advanced AML are both diseases where genetic and epigenetic changes interact to promote initiation and progression of the cancer phenotype [17]. In approximately 30% of these patients, VPA induces pronounced cytostatic effects, disease stabilization and promising hematological responses [13]. Hence, identification of resistance mechanisms and effective co-therapeutics are important in order to improve VPA-efficacy in the non-responsive patients.

Epigenetic changes in cancer are global and a large number of enzymes are known to covalently modify histones and DNA with varying effects on different genes [1]. Given this complexity, there is lack of clarity of mechanisms and interrelation between different types of histone marks and the enzymes that deposit them. Appropriate functional genomic strategies are well suited to analyze the global biological end-points of such wide-ranging responses [17]. As changes in transcription are direct biological end-points of epigenetic reprogramming we previously identified gene sets unique for AML cells from VPA resistant patients [14]. The functional and mechanistic relevance of the gene expression changes were difficult to determine as different processes mediating epigenetic regulation of gene expression are intimately linked and affect a range of biological endpoints. Proteomic approaches are therefore used to supplement gene expression analyses and have been successfully implemented in the identification of new targets for improvement of conventional chemotherapy in AML [18], [19], [20]. Another approach to identify appropriate anti-cancer epigenetic switches is genetic interaction-studies to identify synthetic lethal interactions. Synthetic lethal interactions may also identify prognostic markers and mechanistic requirements of drug action.

*Caenorhabditis elegans (C. elegans)* is a powerful animal model for assessment of functional roles of genes and pathways [21], [22]. Robust RNA interference (RNAi) technology contributes to the success of *C. elegans* by allowing synthetic lethality screens to be performed [23]. RNAi may also provide a highly effective method for discovery of therapeutic targets in AML [24], [25]. Moreover, *C. elegans* is an appropriate model to assess functions of VPA-regulated genes; VPA induces similar responses in *C. elegans* as in mammalian cells, including activation of DNA damage response [26] and developmental arrest.

**Figure 1:**
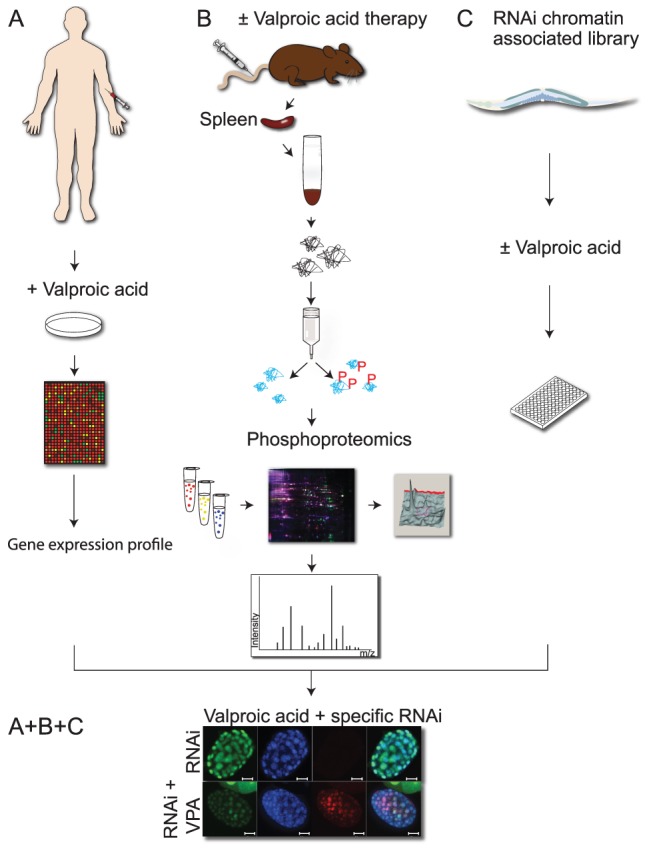
Gene expression analysis, phosphoproteomics and *C. elegans* chemical-genetic screen identify conserved responses to valproic acid. **A**) Human primary AML blasts were treated with 600 µM valproic acid (VPA), resulting in the discrimination of responsive and non-responsive cells to VPA by gene array expression studies [14]. **B**) Leukemic BNML rats were treated with vehicle or VPA (170 mg/kg *b.i.d.*). Phosphorylated proteins were collected from leukemic blasts from the spleen *post mortem* and separated by DIGE. Differentially represented phosphoproteins in animals treated with VPA were identified by Orbitrap mass spectrometry. **C**) The Ahringer chromatin-associated gene library was combined with 15 mM VPA for 48 hours and screened for synthetic lethality defined by developmental arrest. **A+B+C**) Functional validation of targets from all three screens (A, B and C) by combining RNAi with VPA (15 mM) in *C. elegans.* The effect on acetylation for chosen targets was investigated by immunofluorescense in *C. elegans* embryos (lower panel).

We hypothesized that use of *in vivo* models for functional validation would facilitate the translation of complex datasets into clinically useful biomarkers and molecular targets for enhancement of VPA-therapy in AML at low cost. A pre-existing human gene expression dataset of VPA resistance was complemented with an *in vivo* rat leukemia phosphoproteomic screen, and synthetic lethality in *C. elegans* was exploited as a functional validation tool (Figure 1). Using this strategy we identified novel conserved sensitizers and synthetic lethal interactors of VPA, as well as conserved resistance pathways converging on HSP90AB1, HSP90AA2, and MAPKAPK2. These observations, together with a functional relationship between protein acetylation and protein methylation involving UTX (UTX-1) suggested multiple molecular mechanisms for effective anti-cancer valproic acid therapy.

## Materials and Methods

### Animals

200–250 g male Brown Norwegian rats (BN/mcwi) (Charles River Laboratories, Wilmington, MA, USA) were injected intravenously in the lateral tail vein with 10 million (pulsed treatment (PT) group) or 5 million (chronic treatment (CT) group) Brown Norwegian myeloid leukemia (BNML) cells on day 0 respectively. The PT group received VPA (Desitin Pharma AS, Hamburg, Germany) by intra peritoneal injections (400 mg/kg) and the CT group by oral gavage (170 mg/kg). The control group received vehicle only. Treatment was initiated day 10 (PT) or day 16 (CT) increasing the dose on day 17 (170 mg/kg twice daily (*b.i.d.*)) for the latter group. Animals were treated until sacrificed at humane endpoint, defined as loss of 10–15% of body weight in addition to ataxia, paralysis of hind or fore limbs, lethargy or dehydration. Survival ratios were investigated by performing the Log-rank (Mantel-Cox) Test on Kaplan-Meier curves. All animal experiments were reviewed and approved by The Norwegian Animal Research Authority under study permit number 2004 190, and conducted according to The European Convention for the Protection of Vertebrates Used for Scientific Purposes.

### Harvesting of BNML cells and phosphoproteins

Spleens were excised, segmented and diluted with 0.9% NaCl. The filtered solution (40 µm Nylon Cell Strainer (BD Biosciences, Franklin Lakes, NJ, USA)) was homogenized prior to isolation of leukocytes by density gradient separation by Lymphoprep as described by the manufacturer (Nycomed Pharma Diagnostics, Asker, Norway).

Phosphorylated proteins from 20 million BNML blasts were harvested using the PhosphoProtein Purification Kit (Qiagen, Hilden, Germany) according to the manufacturer's description. Phosphorylated proteins were recovered after immobilized metal-affinity chromatography (IMAC). Following concentration by trichloroacetic acid precipitation the washed pellets were resuspended in difference gel electrophoresis (DIGE) sample buffer (GE Healthcare, Little Chalfont, UK) and frozen at −80°C

### 2D DIGE

Phosphoprotein samples were covalently labeled with fluorescent CyDyes (GE Healthcare) in a minimal labeling reaction as described previously [27] with minor modifications; pooled, labeled phosphoprotein samples were cup-loaded on pH 3–11 DryStrip Immobiline gel strips (GE Healthcare) prior to isoelectric focusing at 150 V Step 3 hours, 300 V Step 3 hours, 1000 V Gradient 6 hours, 8000 V Gradient 2 hours, 8000 V Step 3 hours. Focused strips were further equilibrated (6 M urea, 2% SDS, 50 mM Tris-HCl, pH 6.8, 30% glycerol) supplemented with 15 mg/ml dithiothreitol for 15 min at room temperature, followed by 45 mg/ml iodoacetamide for 10 min. Second dimension gel electrophoresis was performed on 26×20 cm 10% Ettan DALTsix gels casted in lab and run as described by manufacturers. Gels were run at 6 W overnight, increasing the power to 100 W at the end of the run. Preparative gels were dyed with SYPRO Ruby gel staining (Bio-Rad, Hercules, California, USA) over night and scanned by the Typhoon TRIO Variable Mode Imager (GE Healthcare). Gels were stored in 10% ethanol at 4°C until automatic spot picking by Ettan Spot Picker (GE Healthcare).

### Software analysis of phosphoproteins

Gel images were analyzed using DeCyder 6.5 software (GE Healthcare). Briefly, phosphoprotein spots were co-detected and quantified in the Differential In-gel Analysis module. Protein statistics (unpaired t-test, p<0.05) was performed in the Biological Variation Analysis module, excluding proteins present in less than 80% of the spotmaps. SyproRuby stained single gels spots were in-gel digested for 16 hours prior to peptide identification using the LTQ-Orbitrap XL (Thermo Scientific, Waltham, MA, USA) as described [28]. A minor adjustment was made increasing the starting solvent from 0 to 7% B.

### Pharmacokinetic study of VPA

280–320 g BN rats were injected with 400 mg/kg VPA (Orfiril, injection fluid, Desitin Pharma AS) intra peritoneal at hour 0, or administered 170 mg/kg VPA (Orfiril, oral mixture, Desitin Pharma AS) orally at hours 0 and 8. Blood samples were collected from the tail vein at hours 0, 1, 2, 4, 8 and 24 for the QD regimen, and additionally at hours 9, 10 and 12 for the *b.i.d.* regimen. Serum was collected by incubation for 30 minutes prior to centrifugation at 10 000 rpm for 10 minutes. Serum concentration of VPA was measured by the Laboratory for Clinical Biochemistry at Haukeland University Hospital according to the producer's recommendations, using the CEDIA Valproic Acid II Assay (Microgenics, Thermo-Fisher Scientific, Waltham, MA, USA) on the Modular Analytics System (Roche Applied Science, Inc., Penzberg, Germany). Steady state levels of the drug were calculated based on 4 and 5 half-lives of VPA.

### *C. elegans* strains and culture conditions

*C. elegans* strains wild type Bristol N2, RNAi sensitive NL2099 *rrf-3*(pk1426), MT14480 *set-11*(*n4488*) II, NU1 *bra-1*(*nk1*) X, VC1213 *gei-8*(*ok1671*) III, MH2430 *cbp-1*(*ku258*) III and AZ212 *unc-119*(*ed3*) ruls32[*unc-119*(+) *pie-1*::GFP::H2B] III (*Caenorhabditis elegans* Genetic Center, University of Minnesota, USA) expressing GFP in fusion with H2B, as well as the mutant strains *utx-1(3136)* rescued with puts-1 *UTX-1*::GFP and C29F7.6 (*jmjd-3.*3) (kind gift from Dr. Lisa Salcini, BRIC, University of Copenhagen) were cultured using standard procedures at 20°C. RNA interference (RNAi) mediated depletion of genes present in the 257 gene large chromatin RNAi library (Source Bioscience Geneservice, Nottingham, UK) was performed by feeding in liquid culture with *E. coli* HT115(DE3) expressing double stranded RNA (dsRNA) from the plasmid vector L4440. The bacteria were grown overnight at 37°C in 600 µl LB medium including 50 µg/ml Carbencillin, induced with 4 mM IPTG at 37°C for one hour, pelleted and resuspended in 100 µl M9 buffer.

### Synthetic lethality in *C. elegans*

*C. elegans* orthologs were identified through Wormbase or by Blast searches. Two parallel screens were performed in the reference *C. elegans* wild type N2 strain and in the RNAi sensitive *rrf-3* mutant. A pilot study was performed to identify the VPA concentration that allowed the identification of synthetic lethal RNAi clones, as those giving severely arrested development in the presence of VPA, and VPA-sensitizers as those that relieved or suppressed the developmental arrest caused by VPA alone. Approximately 20 L1 larval stage worms were dispensed per well in 96-well flat-bottomed tissue culture plates containing 50 µl freshly induced bacteria. Plates were incubated with shaking at 20°C for 24 hours prior to addition of 15 mM VPA. Phenotypes were scored from 0–4 for developmental arrest 72 hours after RNAi exposure. 0 was defined as basal level arrest observed in untreated control worms, 1; worms arrested at L4, 2; arrested at L2-3, 3; arrested at L1 and 4; very few surviving L1 larvae. Positive hits were defined as those giving the same phenotype in 2 out of 3 experiments, in one or both strains. RNAi resulting in high levels of developmental arrest not reversed or increased by VPA were excluded from the study. The RNAi screen was validated using available mutants; approximately 50 L1 larva of the strains N2, *set-11*, *bra-1*, *gei-8*, *cbp-1*, *utx-1*and *jmjd-3.3* mutant dispensed in M9 buffer including *E.coli* OP50 for 24 hours at 20°C prior to treatment with 0 mM, 1 mM or 5 mM VPA. After 72 hours of exposure to VPA the phenotypes were scored for survival of adults. Survival was scored and presented as the percentage of L1 that developed into adults after 72 hours from two independent replicates with at least 50 to 100 animals per data point.

### *C. elegans* immunofluorescense

N2 or AZ212 L4 worms were subjected to RNAi and the mutant strain *utx-1(3136)* was fed with *E. coli* OP50 for one hour prior to co-exposure to 15 mM VPA for 24 hours. Gravid adults were dissected and embryos were transferred to polylysine-coated slide and frozen on dry ice for 20 minutes prior to fixation at −20°C for 10 minutes with methanol followed by acetone. Embryos were immunostained using the primary antibody Acetyl-Histone H4 (Lys8) 1∶1500, primary antibody Di-Methyl-Histone H3 (Lys36) 1∶1500 (both Cell Signaling Technology, Inc., Beverly, MA, USA), and secondary antibody donkey anti-rabbit alexa-555 1∶1500 (Invitrogen, Carlsbad, USA) as described [29]. DAPI (200 ng/ml) (Invitrogen) was included for DNA staining prior to visualization using a LSM-510 META^MK14^, 63×/1.4 oil objective Plan-Apochromat (Carl Zeiss MicroImaging GmbH, Goettingen, Germany), using the AxioVision Version 4.8.2 software. The degree of acetylation and methylation was scored as the percentage of 100-cell stage embryos with positive staining in two independent replicates with at least ten embryos per data point.

### Human AML cell culture and cell death assay

Cell lines were grown in RPMI 1640 (Gibco, Invitrogen, Paisley, UK) (MV4-11 in IMDM (Gibco, Invitrogen)) supplemented with 10% fetal bovine serum gold (PAA Laboratories GmbH, Pasching, Austria), streptomycin (5 mg/ml), penicillin (5 U/ml), and L-glutamine (2 mM) (all from Sigma-Aldrich, St. Louis, Missouri, USA) at 37°C with 5% CO_2_ in a humidified incubator. Wild type *UTX* cell lines MV4-11 and NB4, and UTX mutant cell lines MONO-MAC-1 and THP-1 (all from Deutsche Sammlung von Mikroorganismen und Zellkulturen GmbH (DSMZ), Braunschweig, Germany) were incubated for 48 hours in a 96-well microplate (2×10^4^ cells/well) after treatment with 2 mM VPA (Desitin Pharma AS). Cells were stained with 10 µg/ml Hoechst 33342 DNA stain (Calbiochem, Merck KGaA, Darmstadt, Germany) for one hour at room temperature prior to examining nuclear morphology by epifluorescence microscopy (Leica DM IRB, Leica Microsystems, Mannheim, Germany). Cells were imaged immersed in 1∶1 solution of Fluoro-gel II containing DAPI (Electron Microscopy Sciences, Hatfield, PA, USA) using the Axio Imager.Z1, 100×/1.4 oil objective Plan-Apochromat and the AxioVision Version 4.8.2 software (all by Carl Zeiss MicroImaging GmbH, Goettingen, Germany). Statistical significance was determined using an unpaired, two-tailed t-test (GraphPad, GraphPad Software, Inc., La Jolla, CA, USA). MOLM-13 (DSMZ), harboring functional p53 resembling the majority of the primary patient material [30], were added 1 or 2 mM VPA or 0.2 mM SAHA (kindly provided by Sigrid Rasmussen, Merck Sharp, Whitehouse Station, NJ, USA) dissolved in Dimethyl Sulfoxide (DMSO) (Lab-Scan Analytical Sciences, Gliwice, Poland), 5 nM Geldanamycin (Sigma-Aldrich) dissolved in DMSO, 5 µM Cytochalasin B (Sigma-Aldrich) dissolved in DMSO or 1 nM Vincristine (Pfizer, New York, NY, USA), or a combination of VPA and Geldanamycin, Cytochalasin B or Vincristine, or SAHA and Geldamamycin. Cells were incubated for 48 hours and scored for abnormal nuclei. Statistical significance of drug interaction was determined using two-way ANOVA (GraphPad, GraphPad Software, Inc.). MV4-11 and NB4 cells were treated with 1 mM VPA in combination with 5 nM 17-dimethylaminoethylamino-17-demethoxygeldanamycin (17-DMAG) (Infinity Pharmaceuticals, Cambridge, MA, USA) dissolved in DMSO, 0.5 µM vincristine or 2 µM Cytochalasin B for 48 hours. Viability was determined using the Annexin-V Alexa Fluor 488 (Life Technologies Ltd, Paisley, UK) and Propidium Iodide (PI) (Sigma-Aldrich) assay. Cells were washed in PBS and re-suspended in binding buffer (2.5% Annexin-V Alexa Fluor 488). Samples were incubated for 15 minutes at room temperature and added binding buffer with PI (final concentration 0.2 µg/ml). The data were acquired on a BD Accuri C6 flow cytometer (BD Bioscience, San Jose, CA, USA) and analyzed using the software Flow Jo (Tree Star, Inc., Ashland, OR, USA). The percentage of dead cells is displayed relative to untreated control cells. Statistical significance was determined using two-way ANOVA (GraphPad, GraphPad Software, Inc.) for synergism testing (*p*<0.05).

### Western blotting

5–10×10^6^ cells were washed in ice cold 0,9% NaCl and subjected to gel electrophoresis (10%) and western blotting as previously described [31]. Anti-H3K27me3 (Active Motif, Rixensart, Belgium) and anti-H2BK120ac ((07-564) Upstate Cell Signalling Solutions) were incubated for 1 hour at room temperature, anti-EZH2 ((D2C9) Cell Signaling Technologies, Billerica, MA, USA), and anti-GST-UTX (kindly provided by Prof. Kristian Helin [32]) was incubated overnight at 4°C. Anti-beta-actin ((sc-2778) Santa Cruz Biotechnology, Santa Cruz, CA, USA) was used as loading control. IgG secondary antibodies were from Jackson ImmunoResearch (West Grove, PA, USA). Bands were quantified using the Kodak analysis software (Eastman Kodak Co, Rochester, NY, USA). Data were exported to an Excel spreadsheet, corrected for background and loading control intensities. The mean intensity of a representative Western blot was calculated and normalized to beta-Actin. The numbers shown are arbitrary units compared to the intensity of the MV4-11 control or non-specific siRNA control.

### siRNA knockdown

MV4-11 cells with intact p53 signalling, and p53 mutated NB4 cells were cultured as described above. Medium was replaced with penicillin-free medium the day before the experiment. siRNA knockdown was performed three times in triplicate using the Neon Transfection System according to the suppliers recommendations (Invitrogen). Briefly, 0.2×10^6^ cells, or 1×10^6^ cells for western blot, were electroporated with ON-TARGETplus SMARTpool UTX siRNA (Thermo Scientific, Inc. for MV4-11 cells), UTX siRNA (Qiagen, Inc. for NB4 cells), or negative control (AllStars Negative Control Alexa Fluor 488 siRNA, Qiagen) prior to plating in a 96 or 6 well plate, respectively, at a total concentration of 600 nM. 2 mM VPA was added after 18 hours and cells were incubated for additionally 48 hours, prior to scoring for abnormal nuclei as described above. For Western blotting, cells were incubated for 24 hours with negative control or UTX siRNA at a total concentration of 600 nM prior to cell lysis and gel electrophoresis as described above. Statistical significance of drug and siRNA interaction was determined using an unpaired, two-tailed t-test (GraphPad, GraphPad Software, Inc.).

### Small-scale data integration

Data from all screens were combined *in silico* for extraction and prediction of common functionalities and components; Lists of direct protein-protein interactors as well as indirect interactors mediated via one neighbors were extracted using FunCoup (Stockholm Bioinformatics Centre, http://funcoup.sbc.su.se) [33]. Next, the results from each individual screen were combined to find common hits across the different screens. The lists were imported into Cytoscape (http://www.cytoscape.org) [34] in order to find enriched Biological Processes using the plug-in program BiNGO (Flanders Interuniversitary Institute for Biotechnology, http://www.psb.ugent.be/cbd/papers/BiNGO/Home.html) [35]. False discovery rate was controlled by the Benjamini-Hochberg procedure, correcting the p-values for functional coupling of the proteins.

## Results

### Genes induced by VPA in non-responsive AML patient cells reflect resistance mechanisms

We previously identified genes up-regulated in response to VPA (Table S1; *ABCA5*, *AGPAT4*, *BAG2*, *COCH*, *FLIPT1*, *WDR35*, *EID3*, *KCNA3*, *MAD1*, *SERPINF1*, *SMAD3*, *AKT3*, *IL12RB2*, *NDRG2*) in AML cells isolated from responding and non-responding patients *in vitro* [14], and hypothesized that AML cells proliferating in the presence of VPA (non-responsive) induced genes that contributed to resistance. To test this hypothesis we assessed whether depletion of these genes exacerbated or suppressed the developmental arrest phenotype induced by VPA in *C. elegans* (Figure 1).

Synthetic lethal interactions were defined as a combination of VPA and RNAi that led to arrest at earlier developmental stages than by either treatment alone. Conversely, if genes up-regulated in the responding primary cells reflect a mechanistic function we would expect to see RNAi-induced suppression of the VPA-induced phenotype – such genes were therefore defined as sensitizers of VPA: No such sensitizers of VPA were found. Contrary to this, we found that depletion of orthologs of the six genes that were up-regulated in the VPA-non-responsive cells resulted in synthetic lethality, thereby confirming our hypothesis (Table S1). However, 5 out of 15 genes up-regulated in the responsive cells also resulted in synthetic lethality (Table S1). Thus, genes transcriptionally up-regulated in both non-responding and responding leukemic patient cells may contribute to cell survival in the presence of VPA.

### Establishment of a BNML leukemic rat VPA progressive disease model

**Figure 2:**
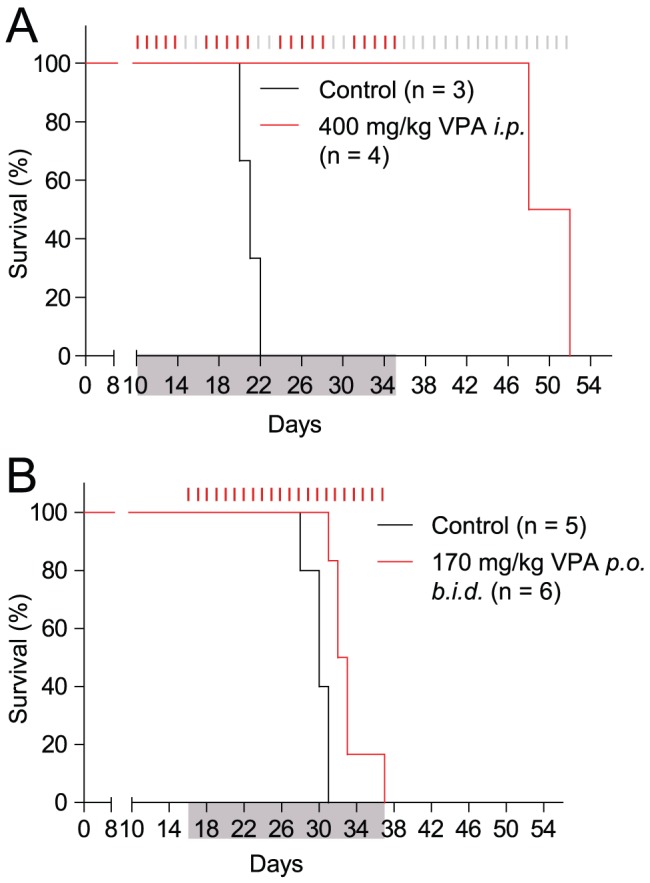
Survival of leukemic BN rats without and with valproic acid treatment. **A**) BN rats were injected with 10×10^6^ BNML cells on day 0 before treatment started on day 10. Animals were treated with vehicle or VPA intra peritoneal (400 mg/kg) for 5 days, with 2 days off, in total for four weeks. Animals did not display symptoms for leukemia until termination of treatment, representing a responsive VPA-model. **B**) BN rats were injected with 5×10^6^ BNML cells on day 0 before treatment (170 mg/kg VPA, per orally) started at day 16. Animals were treated successively with 170 mg/kg VPA *b.i.d.* from day 17. Animals treated with VPA showed significantly increased survival compared with control rats (median survival increased from 30 to 32.5 days, *p* = 0.0037). However, the disease progressed and animals displayed high leukemic burden upon sacrifice at humane endpoint, representing a VPA-resistant model. Red marks represent days of treatment, grey marks represent no treatment.

To further explore the VPA-resistance mechanisms we utilized the Brown Norwegian myeloid leukemia (BNML) syngeneic rat leukemia model which provides highly reproducible responses to conventional chemotherapeutics [18], [36] with the advantage of a complete host immune system during leukemia growth and therapy. We developed a progressive disease model in the BNML rats by giving animals suboptimal doses of VPA (170 mg/kg *b.i.d.*). This dose level partly reflects the clinical situation when VPA-dose is reduced due to adverse effects like drowsiness [13]. The rats presented with a progressive, minimally-responsive disease. Treatment with 400 mg/kg VPA, however, resulted in survival for the duration of VPA therapy in this experiment (4 weeks) (Figure 2A), reflecting the potential of high dose VPA to prevent disease progression. However, lower level dosing resulted in a minimal, yet significant, extension of the median survival from 30 to 32.5 days (*p* = 0.0037) (Figure 2B) similar to the progressive disease often observed in advanced AML patients treated with VPA [13], [37]. Both doses give clinically relevant steady state serum concentrations of 174–361 µM and 250–500 µM for the high and low dose model, respectively (Figure S1). The high dose treatment results in consequently higher VPA serum concentrations during the first eight hours, compared to the low dose. Additionally, the high dose gives higher average area under the curve (AUC) values 1 354 and 880 µM/hour by the linear and logarithmic trapezoidal method, respectively, compared to 372 and 356 µM/hour by the low dose. We therefore believe the peak concentration and the AUC of the drug, and not the steady state levels to be determinant of VPA responsiveness. Death by progressive leukemia was confirmed by necropsy, revealing extensive splenomegaly and hepatomegaly compared to untreated BN rats. Hence, the low dose VPA regime represents an *in vivo* model of progressive leukemic disease on treatment with VPA.

### VPA regulates phosphoprotein expression in progressive leukemic disease

We used phosphoproteomics to identify active signaling pathways induced by VPA in the BNML rat VPA-minimal-response model. Phosphorylated proteins from spleen-derived leukemic cells were collected at the defined disease endpoint, mimicking progressive disease and VPA therapy failure (Figure 1B). Twenty-one differentially expressed phosphoproteins (*p*<0.05) were identified by linear quadrupole ion trap-Orbitrap (LTQ-Orbitrap) Mass Spectrometry (Table 1).

**Table 1:** Valproic acid-modulated phosphoproteins from BNML rat leukemia progressive disease.

	DeCyder Protein Analysis			Synthetic lethality		Gene information	
	Protein	Ratio (VPA/CTR)	p-value	*C. elegans* gene	Synthetic lethal	Gene	Biological process
1	SPG21	−1,97	0,0059	*		*Spg21*	Cell death, CD4 activation
2	PAI-RBP1			*vig-1*	Y	*Serbp1*	Regulation of mRNA stability, regulation of anti-apoptosis
	ATPA1	−1,96	0,05	H28O16.1	Y	*Atp5a1*	Negative regulation of endothelial cell proliferation, ADP
							and ATP biosynthesis
3	HK1	−1,7	0,024	H25P06.1	Y	*Hk1*	Glycolysis
	SHIP1			*		*Inpp5d*	Negative regulation of cell proliferation
4	OPA1	−1,55	0,025	*eat-3*	Y	*Opa1*	Apoptosis, mitochondrial organisation
	DYN2			*		*Dnm2*	Endocytosis, G2/M transition of cell cycle
5	MOESIN	−1,38	0,0043	*		*Msn*	Leukocyte migration and cell-cell adhesion
6	GOT2	−1,33	0,022	*		*Got2*	Cellular amino acid metabolic process, fatty acid
							transportation
7	HPRT1	−1,29	0,032			*Hprt1*	Lymphocyte proliferation, nucleoside metabolism
8	PRPSAP2		0,016	W04G3.5	Y	*Prpsap2*	Nucleoside metabolism, regulation of transcription
	MAPK	1,38		*		*Mapk1*	Induction of apoptosis, regulation of proliferation, DNA damage response
9	HSC70	1,39	0,0083	*hsp-1*	-	*Hspa8*	Unfolded protein response
10	TUBA1B	1,41	0,017	*tba-2*	-	*Tuba1b*	Microtubule cytoskeleton organization
	PPM1F			*		*Ppm1f*	Apoptosis, protein dephosphorylation
11	UQCRC2	1,42	0,046	*ucr-2.2*	-	*Uqcrc2*	Oxidative phosphorylation, proteolysis
12	NAGK	1,45	0,043	*		*Nagk*	N-acetylglucosamine metabolic process
13	NUCB2	1,48	0,034	*		*Nucb2*	DNA binding
14	eEF1D	1,53	0,039	*		*Eef1d*	Translational elongation, positive regulation of I-kappaB
							kinase/NF-kappaB cascade
15	GMFG	1,57	0,037	*		*Gmfg*	Growth factor activity, protein phosphorylation
16	LXN	1,65	0,000077	*		*Lxn*	Enzyme and metalloendopeptidase inhibitor
17	APRT	1,67	0,014	T19B4.3	Y	*Aprt*	Purine salvage, nucleoside metabolism
18	ACTB	1,67	0,043	*act-5*	**	*Actb*	Cellular component movement, axonogenesis
	NUDC			*nud-1*	Y	*Nudc*	Cell proliferation and cytokinesis
19	SET	1,75	0,00075	*		*Set*	Negative regulation of histone acetylation, DNA replication
20	PSME2	1,8	0,00047	*		*Psme2*	Proteasome activator
21	ACTB	1,96	0,019	*act-5*	**	*Actb*	Cellular component movement, axonogenesis
				Control	-		

Positive values of ratios between VPA-treated and control animals indicate proteins with elevated expressed level, negative values are proteins with reduced expression in VPA treated animals.

* RNAi was not performed.

** Synthetic lethality could not be assessed because of severe developmental arrest by RNAi alone.

Among the differentially phosphorylated proteins were eleven that participate, or are predicted to act, in the resistance pathways identified from the gene expression analysis [14] (Figure S2); Phosphoproteins involved in ubiquitin dependent protein degradation, (PSME2 and HSC70), oxidative stress/MAPK signaling (MAPK1, eEF1D), TGFβ signaling (SHIP1), mitochondrial ATP synthesis/oxidative phosphorylation (OPA1, UQCRC2), and intracellular transport (TUBA1B (tubulin), DYN2 (Dynamin 2), ACTB (actin), MOESIN) were differentially represented in the VPA-treated rats. The enrichment of cytoskeletal proteins was consistent with a requirement for the cytokinesis checkpoint for sustained proliferation in the presence of VPA. Functional validation in *C. elegans* showed that synthetic lethality was observed for 7 out of the 11 genes investigated (Table 1). In contrast to the severe toxicity associated with depletion of classical checkpoint proteins, the targets identified here resulted in low or no toxicity in absence of VPA, emphasizing their potential for development as combination therapy.

### Abrogation of conserved resistance pathways sensitizes human AML cells to VPA

Although VPA is a HDACi [38] we found a striking under-representation of genes involved in chromatin remodeling in the above analyses, with SET and NUCB2 being the only DNA binding proteins identified in the phosphoproteomic screen (Table 1). To identify functional interactions between VPA and genes participating in chromatin associated processes, we screened a focused *C. elegans* RNAi library that identified 43 genes that modulated VPA-induced developmental arrest of which an additional 28 synthetic lethal clones were identified, 6 of which are predicted, or known, transcriptional regulators (Table 2, Figure 1C).

**Table 2:** VPA-synthetic lethal or -sensitizer genes identified from the chromatin library screen in *C. elegans*.

Sequence name	*C. elegans* gene	Human gene	Biological process of *C. elegans* gene
**VPA-synthetic lethal genes**			
T27F2.1	*skp-1*	*SNW1*	Chromatin binding and nuclear mRNA splicing
D1081.8	*phi-7*	*CDC5L*	DNA damage response, mRNA splicing factor
F45E1.6	*his-71*	*H3F3B*	DNA damage response, nucleosome assembly
W05B10.1	*his-74*	*H3F3B*	DNA damage response, nucleosome assembly
Y49E10.6	*his-72*	*H3F3B*	DNA damage response, nucleosome assembly
C12C8.3	*lin-41*	*TRIM71*	Predicted E3 ubiquitin ligase, negative regulation of translation
T11A5.1	lys-12	*MYST3*	Histone acetyl transferase
T08D10.2	*lsd-1*	*KDM1A*	Lysine-specific histone demethylase
F34D6.4	*set-11*	*EHMT2*	Putative histone H3 lysine-9 methyltransferase
R06F6.4	*set-14*	*SMYD3*	Predicted histone tail methylase
ZC8.3	*set-30*	*SMYD3*	Predicted histone tail methylase
C14B9.6	*gei-8*	*NCOR1*	Likely homolog of co-repressor NCoR/SMRT
F08G2.2	*his-43*	*HIST2H2AB*	Nucleosome assembly
F08G2.3	*his-42*	*HIST2H3D*	Nucleosome assembly
F17E9.13	*his-33*	*HIST2H2AB*	Nucleosome assembly
F17E9.9	*his-34*	*HIST2H2BF*	Nucleosome assembly
F54E12.5	*his-57*	*HIST2H2AB*	Nucleosome assembly
F55G1.10	*his-61*	*HIST2H2AB*	Nucleosome assembly
F55G1.3	*his-62*	*HIST2H2BB*	Nucleosome assembly
H02I12.7	*his-65*	*HIST2H2AB*	Nucleosome assembly
T10C6.12	*his-3*	*HIST2H2AB*	Nucleosome assembly
T23D8.6	*his-68*	*HIST2H2AB*	Nucleosome assembly
ZK131.10	*his-16*	*HIST2H2AB*	Nucleosome assembly, defence response
F54B11.6	*bra-1*	*ZMYND11*	Negative regulation of TGF-beta receptor signalling pathway
B0019.2	B0019.2	*	Regulation of transcription
F43G9.12	F43G9.12	*C21orf66*	Regulation of transcription
R10E11.1	*cbp-1*	*CREBBP*	Regulation of DNA dependent transcription, positive regulation of RNA polymerase II promoter
F54F2.9	F54F2.9	*DNAJC1*	Predicted unfolded protein binding
**VPA-sensitizer genes**			
C44F1.2	*attf-1*	*GMEB1*	AT hook transcription factor family
F13C5.2	F13C5.2	*BRD2*	Bromodomain containing protein
F37A4.8	*isw-1*	*SMARCA1*	ATP-dependent chromatin remodelling
F11A10.1	*lex-1*	*ATAD2B*	Chromatin structure and transcription
F02D10.7	*set-8*	*WHSC1L1*	DNA damage response
ZC155.2	ZC155.2	*	Protein harbouring core histone domains
D2021.1	*utx-1*	*KDM6A*	Histone H3 di/trimethyllysine-27 (H3K27me2/me3) demethylase
C29F7.6	C29F7.6	*JMJD3*	Putative histone H3 di/trimethyllysine-27 demethylase
F18E9.5	*tag-279*	*UTY*	Histone H3 trimethyllysine-27 (H3K27me3) demethylase
K09F5.5	*set-12*	*SETD2*	Histone-lysine N-methyltransferase activity
R11E3.4	*set-15*	*EHMT2*	Predicted histone H3 (Lys9) methyltransferase
F25C8.2	*amx-3*	*SMOX*	Metabolic process
F22F1.1	*hil-3*	*HIST1H1B*	Nucleosome assembly
Y113G7B.14	Y113G7B.14	*TTF2*	RNA polymerase II transcription termination factor family
C49F5.5	C49F5.5	*EP300*	Regulation of DNA-dependent transcription

* No corresponding human homolog.

**Figure 3:**
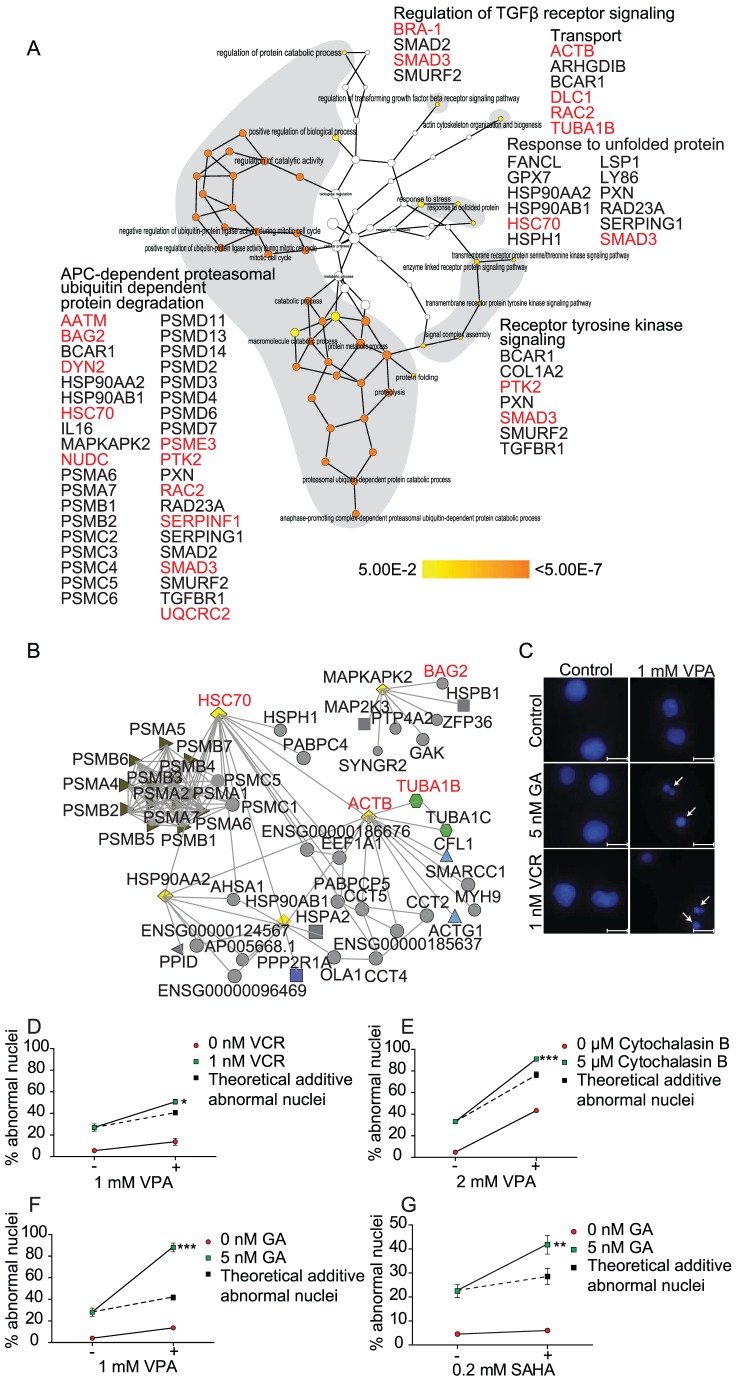
Conserved pathways contribute to VPA resistance. **A**) The human homologs/orthologs of data from all screens were combined in an *in silico* approach to extract and predict common functionalities and components. Proteins identified in our datasets (*red*), as well as direct interactors and indirect interactors (*black*) mediated via one neighbor extracted by the FunCoup browser, were imported into Cytoscape which returned five clusters of enriched process which were manually grouped (grey shade) to illustrate that “APC-dependent proteasomal ubiquitin-dependent protein degradation” and “Response to unfolded protein” are the major conserved processes responding to VPA. In addition, “Regulation of TGFβ receptor signaling” and “Transport” are also functionally important in providing resistance to VPA. The scale depicts color representation of significance by Benjamini-Hochberg correction, where white nodes are not significant, yellow *p*<0.05 and orange *p*<7×10^−7^. **B**) Protein interaction network reveal conserved hubs that promote VPA resistance. Data from all screens were combined *in silico* and the functional interaction network among the common proteins (diamonds) were extracted using FunCoup. ACTB, HSC70, and TUBA1B (*red*) were in the primary list whereas MAPKAPK2, HSP90AA2 and HSP90AB1 (*black*) were identified in all approaches as interactors. ACTB, HSC70, HSP90AA2, HSP90AB1 and TUBA1B represent evolutionary conserved nodes providing resistance to VPA. **C**) VPA (1 mM) was combined with inhibitors of tubulin (vincristine (VCR), 1 nM) or HSP90 (geldanamycin (GA), 5 nM) in MOLM-13 AML cells and investigated for effects on apoptosis measured by Hoechst staining after 48 hours of treatment. Arrows indicate fragmented and condensed nuclei. Scale bar = 10 µm. Combinations of 1 mM VPA and 1 nM vincristine (**D**), 2 mM VPA and inhibitor of actin polymerization cytochalasin B (**E**), 1 mM VPA and 5 nM geldanamycin (**F**), and 0.2 mM SAHA and 5 nM geldanamycin (**G**) all show statistically significant synergism of drug interaction, two-way ANOVA, * *p*<0.05, ** *p*<0.001, *** *p*<0.0001. Error bars represent standard error of mean (SEM).

Although there was no direct overlap between datasets harvested through the different methods used, the individual datasets indicated modulation of similar pathways or biological processes. To extract the common processes reflected in all approaches we analyzed the overlap based on gene ontology (GO) annotation. The biological processes emerging from the three lists show remarkable similarities (Figure 3A). In particular, TGFβ and oxidative stress/MAPK signaling, ubiquitin dependent protein degradation, as well as maintenance of chromatin structure and the cytokinesis checkpoint are conserved processes modulated by VPA. Several of these pathways have been found to be regulated by VPA. The combination of VPA and the proteasome inhibitor bortezomib synergistically increased apoptosis and decreased proliferation in the AML cell line HL60 [39]. Further, genes active in the MAPK, ubiquitin-mediated proteolysis and TGFβ signaling pathways have been found to be up-regulated in response to treatment with VPA and hydralazine in breast cancer patients [40]. Next, we supplemented the primary data with predicted protein-interaction partners extracted using FunCoup [33] to see whether a direct overlap between all methods and model systems could be revealed. As anticipated an overlap emerged for proteins extracted from all models (Figure 3B, *yellow diamonds*). The resulting predicted network includes several primary hits (Figure 3B, *in red*) converging on MAPKAPK2, ACTB, HSP90AA2 and HSP90AB1, suggesting that these are key proteins responding to VPA. Loss of BAG2 (*unc-23*, a direct interactor of MAPKAPK2) sensitized *C. elegans* to VPA (Table S1). We confirmed that other components of this conserved network promote survival in response to VPA also in human cells. Significantly increased VPA sensitivity was seen after concomitant inhibition of tubulin polymerization by vincristine (VCR) (Figure 3C and D), actin polymerization by cytochalasin B (Figure 3E), or HSP90 by geldanamycin (GA) (Figure 3C and F) in the MOLM-13 and the MV4-11 AML cell lines (Figure S3). In the p53 negative AML cell line NB4, synergy was observed by VPA and cytochalasin B treatment (Figure S3). Importantly, potentiation of cytotoxicity beyond the theoretical additive effect by GA was found also when combined SAHA in MOLM-13 (Figure 3G). Hence, the conserved resistance program identified for VPA is valid also for other clinically useful HDACi.

### A synthetic lethal screen in *C. elegans* identify histone demethylases as sensitizers of VPA-induced developmental arrest

Unlike gene expression arrays and phosphoproteomic screens, the *C. elegans* RNAi screen also revealed a high number of genes (15 out of 48) which suppressed the developmental arrested phenotype of VPA when depleted by RNAi (Table 2). These genes were termed VPA-sensitizers as they likely mediate VPA arrest. That five out of 15 sensitizers are known, or predicted to have histone methyltransferase or demethylase activity, suggests that the hyperacetylated state induced by VPA requires additional changes in chromatin state to induce cytostatic effects. All sensitizers, apart from *set-8* and *amx-3*, have DNA binding activity. We conclude that extensive epigenetic chromatin modification is required for cytostatic effects of VPA.

Given the complex regulation of histone marks, it was not unexpected that genes encoding histone methylation (*set-11 (EHMT2)*, *set-12 (SETD2)*, *set-15 (EHMT2)* and *set-30 (SMYD3)*) as well as histone demethylation (C29F7.6 (*JMJD3*), *utx-1 (KDM6A)* and *tag-279 (UTY)*) activities were recovered as both sensitizers and synthetic lethal interactors. Furthermore, we used available *C. elegans* mutant strains of VPA synthetic lethal- as well as sensitizer-genes to validate the RNAi screen and found that mutants of sensitizer genes showed decreased sensitivity to VPA, and conversely, mutants in genes identified as synthetic lethal interactors showed increased VPA sensitivity (Figure S4). In light of the results from the gene expression array and the phosphoproteomic screen, it is intriguing that depletion of a negative regulator of TGFβ signaling, *bra-1 (ZMYND11)* increases the effect of VPA treatment. BRA-1 was suggested to link TGFβ signaling with chromatin remodeling [41]. Hence, the ability of VPA to modulate chromosome dynamics and epigenetic histone marks appear central to the cytostatic effect of VPA.

### Hyperacetylation of H4K8 correlates with the developmental arrested phenotype in *C. elegans*

**Figure 4:**
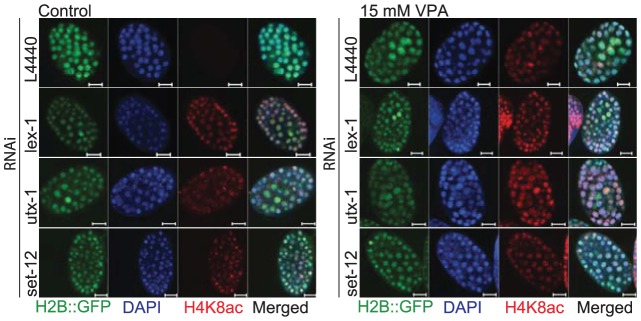
Histone methylation capacity affects basal histone acetylation in *C. elegans* embryos. VPA-treatment induced global acetylation in 100-cell stage *C. elegans* embryos. The strain AZ212, expressing GFP in fusion with H2B, was fed the empty vector L4440 (RNAi), the VPA-sensitizers *lex-1* (RNAi), *utx-1* (RNAi) (histone demethylase), and *set-12* (RNAi) (histone methyltransferase), and exposed to 15 mM VPA at L4 larval stage for 24 hours at 20°C. The embryos were fixed with acetone and methanol prior to staining with an Acetyl-Histone H4 (Lys8) antibody. At the 100-cell stage, baseline levels of acetylation were seen in untreated control worms. Global hyperacetylation was observed after treatment with VPA by all. Depletion of *lex-1*, *utx-1* and *set-12* gives increased baseline acetylation in the absence of VPA. Scale bar = 10 µm.

As a direct read-out of VPA action we monitored the acetylation status of Histone 4 Lysine 8 (H4K8ac), considered to be enriched in transcriptionally active chromatin and independent of DNA damage [42]. In VPA-treated animals, H4K8ac was readily detected at the 100-cell stage (Figure 4) whereas strong H4K8ac staining was first seen approximately at the 200-cell stage in untreated controls (Figure S5A). Hence, as expected from an HDACi, VPA induces a state of histone hyperacetylation in the early embryo and H4K8ac can be used to test whether VPA-regulated genes and proteins directly affect histone acetylation *in vivo*.

Whereas no change was seen in global H4K8ac levels after depleting two synthetic lethal interactors (*oct-2 (FLIPT1)* and *vig-1 (SERBP1)*, data not shown), depletion of all three VPA-sensitizers tested (*set-12 (SETD2)*, *utx-1 (UTX)*, and *lex-1 (ATAD2B)*) gave increased baseline staining for H4K8ac (Figure 4). This suggests that these gene products may interfere with the proper function of HDA-1 (HDAC1) deacetylation activity in early embryos and that elevated histone acetylation levels increases the sensitivity to HDACi.

### UTX mutated cells display reduced VPA sensitivity

To validate that the sensitizers identified in *C. elegans* had a similar function in human AML cells, we focused on UTX as *UTX (utx-1)* mutations have been found in the AML cell lines MONO-MAC-6, THP-1 [43], and in patients with myelodysplastic syndrome [44]. If UTX was a sensitizer of VPA responses, we would predict that it contributes to regulate genes that are activated by VPA. Subsequently, we found several promoters of genes identified as differentially regulated by VPA in human AML patients [14] were consistently occupied by UTX in chromatin immunoprecipitation experiments [45], and human orthologs of synthetic lethal interactors identified here were bound by UTX (Table S2).

**Figure 5:**
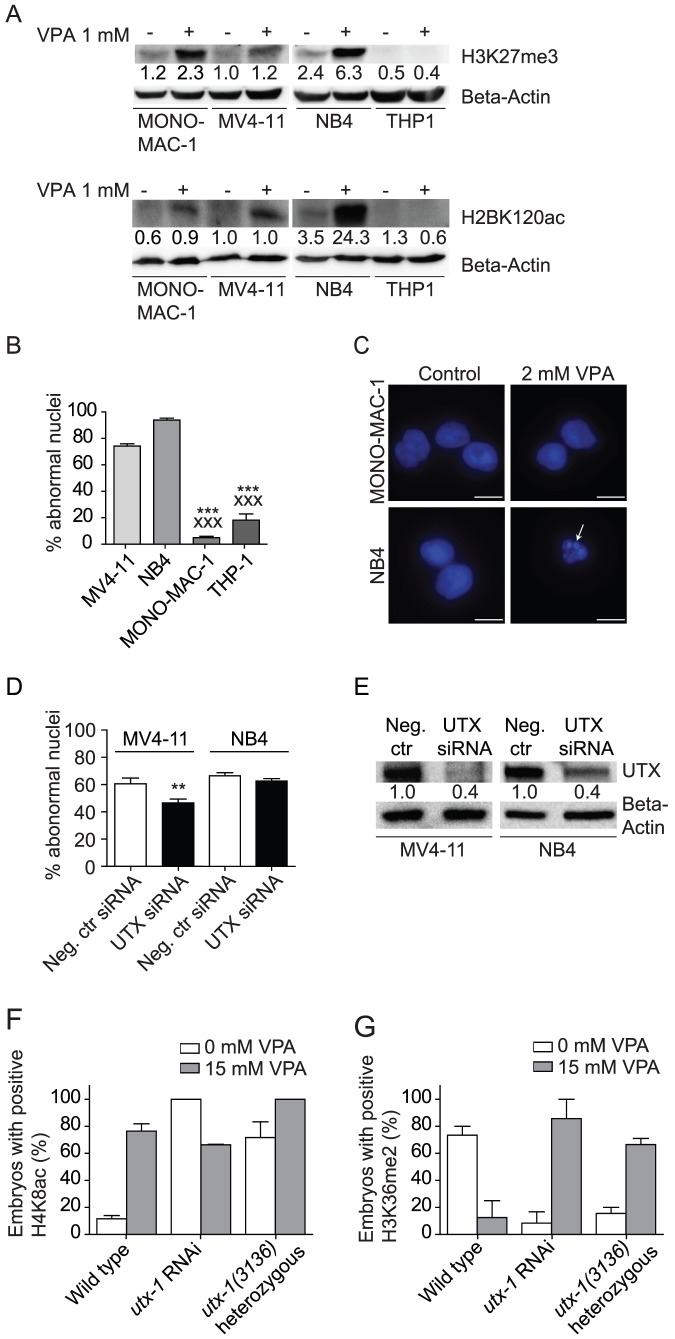
UTX-1 is required for VPA action in *C. elegans* embryos and in human cells. **A**) Two *UTX* wild type cell lines (MV4-11 and NB4) as well as the *UTX* mutant cell line THP-1 were treated with 1 mM VPA for 48 hours and analyzed for H3K27me3 and H2BK120ac expression. The mean intensity on one representative Western blot was calculated and normalized to beta-actin. The numbers shown are in arbitrary units compared to MV4-11 control. Blots show the increase of H3K27 trimethylation as well as an increase in the degree of H2BK120 acetylation except THP-1, where the level of methylation is unchanged and level of acetylation is decreased. **B**) AML cell lines with mutated *UTX* are resistant to VPA. MV4-11 and NB4 as well as two *UTX* mutant cell lines (MONO-MAC-1 and THP-1) were subjected to 2 mM VPA for 48 hours and scored for abnormal nuclei by Hoechst staining. * indicates t-test versus MV4-11, x indicates versus NB4. ***/xxx *p*<0.0004. Error bars represent SEM. **C**) The mutant cell lines show decreased apoptosis, determined by Hoechst staining compared to the wild type cells. Arrows indicate fragmented and condensed nuclei. Scale bar = 10 µm. **D**) MV4-11 or NB4 cells were subjected to 600 nM UTX siRNA or negative control siRNA for 18 hours prior to 48 hours treatment with 2 mM VPA. Cells were scored for abnormal nuclei by Hoechst staining, showing UTX siRNA to reduce the effect of VPA on cell death. Values are normalized against untreated cells. ** indicates t-test of UTX siRNA versus negative control siRNA, *p* = 0.014. Error bars represent SEM. **E**) Western blot of MV4-11 or NB4 cells treated with 600 nM negative control siRNA or UTX siRNA for 24 hours. Numbers shown are arbitrary units compared to negative control siRNA. The blot shows a 60% decrease in UTX by siRNA treatment, confirming the efficacy of transfection. **F**) The *C. elegans* strain AZ212 was fed the empty vector L4440 or *utx-1* (RNAi), and the *utx-1(3136)* mutant was fed *E. coli* OP50 and exposed to 15 mM VPA at L4 larval stage for 24 hours at 20°C. The embryos were immunostained using antibodies recognizing H4K8ac. In the 100-cell stage embryos, baseline levels of this activating acetylation mark were observed in untreated control worms, whereas the *utx-1* (RNAi) and *utx-1(3136)* mutant worms showed highly increased acetylation levels (100% and 76% acetylation, respectively). Error bars represent SEM. **G**) Worms were treated as in F) and stained with a H3K36me2 antibody. At the 100-cell stage, control embryos showed 73% methylation while both *utx-1* (RNAi) and the *utx-1(3136)* mutant showed baseline methylation. By VPA treatment, control worms show baseline methylation whereas both *utx-1* (RNAi) and *utx-1(3136)* mutant shows 86% and 67% methylation respectively. Error bars represent SEM.

The association between histone acetylation and methylation observed in *C. elegans* was also evident in a panel of AML cell lines, including THP-1 cells harboring a deletion comprising exons 1–16 in the *UTX (utx-1)* gene and no detectable protein expression [43]. The expected increase in histone acetylation after VPA treatment was observed in MV4-11 and NB4 cells concomitant with increased H3K27me3 (Figure 5A). Neither histone mark was induced by VPA in the UTX-null line THP-1, consistent with the hypothesis of UTX being contributing to VPA function. A comparison of VPA sensitivity in human AML cell lines demonstrated that two cell lines harboring mutated *UTX (utx-1)*, namely THP-1 and MONO-MAC-1, were significantly less sensitive to VPA-induced cell death (Figure 5B and C), revealing UTX as a sensitizer of VPA toxicity also in human AML cells. A mechanistic requirement for UTX for VPA-response was further confirmed by knock down of UTX by siRNA in the highly VPA-sensitive MV4-11 cell line (Figure 5D) (60% knockdown efficiency), resulting in significantly reduced VPA-induced cell death (*p* = 0.014) in cells depleted for UTX prior to exposure to 2 mM VPA compared to cells receiving non-targeting siRNA control. UTX siRNA knock down in the p53 mutated cell line NB4 (60% knock down efficiency) resulted in slightly reduced sensitivity towards VPA (Figure 5D) suggesting that UTX is a conserved sensitizer of VPA induced hyperacetylation and cytotoxicity. Furthermore, *C. elegans* embryos treated with *utx-1* (*UTX*) RNAi or being heterozygous of *utx-1* showed reverse H4K8ac and H3K36me2 responses to VPA compared to wild type AZ212 worms. This result could indicate that *UTX* affects the epigenetic regulation by VPA not only directly through H3K27 trimethylation, but also through general histone demethylation and its activities as an HDACi.

## Discussion

In order to identify genes and proteins that mediate resistance to the HDACi VPA in AML patients, we used a novel combination of models and technology that allowed us to address the mechanism of VPA induced toxicity at multiple levels. First, as an HDACi, VPA is expected to affect gene expression (VPA-regulated genes in AML patients) [14]. Second, this would lead to changes in cellular signaling pathways (probed by an *in vivo* rat leukemia phosphoproteomic screen) and off-target mechanisms that affect the specific biological endpoint, namely VPA sensitivity or resistance (*C. elegans* functional validation). Bioinformatic data-integration helped us identify a small set of conserved genes and pathways that were functionally validated in human cell lines to be VPA-sensitizers or to promote VPA resistance. We propose this to be a powerful strategy to facilitate the translation of complex datasets into clinically useful biomarkers and therapy targets for VPA.

Whereas all experimental approaches successfully revealed genes that confer resistance to VPA, the *C. elegans* chemical genetic screen also revealed mechanistically important genes required for VPA action where a direct modification of the chromatin acetylation state by VPA was demonstrated. Interestingly, several histone demethylases and methyltransferases were required for VPA induced developmental arrest. In embryos depleted for these VPA-sensitizers, the global histone acetylation levels were elevated even in the absence of VPA (Figure 4), suggesting that the sensitizers directly or indirectly restrict histone acetylation. Interestingly, the histone acetylation state was elevated to a similar degree by the two methylation-modulating proteins UTX-1 (UTX), a histone H3 lysine 27 (H3K27) di/trimethyl demethylase and SET-12 (SETD2), a histone methyltransferase. This may suggest that removing either activity will result in destabilization of the methylation pattern of histone H3, permitting access to chromatin for HATs. The recent demonstration that human UTX associates with the H3K4me3 histone methyltransferase MLL2 [45] supports a model in which the coordinated removal of repressive marks and deposition of activating marks are important for regulation of transcription during cellular differentiation. The complex inter-regulation of different histone marks is illustrated in *C. elegans* where VPA treated embryos was depleted of H3K36me2 (Figure S5B) and in human cells by the increase in histone H3K27me3 in response to VPA (Figure 5A, Figure S6). A role for UTX as a sensitizer of cellular responses to VPA was also confirmed in human cells where two human AML cell lines with impaired UTX function showed VPA resistance (Figure 5B). The UTX loss of function mutant cell line THP-1 did not display similar histone modification changes in response to VPA as seen in UTX-proficient AML cell lines (Figure 5A). Furthermore, the depletion of *UTX (utx-1)* in the highly VPA-sensitive cell line MV4-11 resulted in a significant (*p* = 0.014) decrease in cell death when subjected to VPA (2 mM) (Figure 5D). This confirms a role of UTX in mediating the VPA response. Hence, UTX (also known as *KDM6A*) -mediated histone di/trimethyl demethylation results in a more open and active chromatin conformation [46], is here revealed as a requirement for HDACi function. As such, functional *UTX (utx-1)* expression may serve as a biomarker for optimal therapeutic effect of this drug in patients. As an interesting parallel, over-expression of a related histone lysine demethylase, KDM5A, was recently shown to be a possible mediator of broad-spectrum drug-tolerance [47]. It was proposed that altered regulation of chromatin structure or histone marks was an underlying mechanism of reversible tolerance to a wide spectrum of anti-cancer drugs, an observation supported by this study. However, the synthetic lethal interactors identified here show that the increased histone acetylation resulting from inhibition of HDAC class I and II by VPA can, to some degree, be compensated for by activation of tolerance programs.

Our data is further corroborated by clinical trials identifying the TGFβ pathway to be regulated by VPA in breast cancer [12] and reports of deregulated TGFβ signaling in leukemogenesis [48]. A role of TGFβ signaling in response to VPA was reflected in all our datasets and identification of BRA-1 (ZMYND11), a negative regulator of TGFβ signaling in *C. elegans*, as a synthetic lethal interactor provides support for direct regulation of TGFβin addition to an up-regulation of TGFβ negative regulators via AKT inhibition as suggested previously [49].

Increased PAI-RBP1 (the protein of the gene *SERBP1 (vig-1)*) expression was found in chronic lymphocytic leukemia [50] and correlated with tumor progression in epithelial ovarian cancer [51]. Our results points to a possible function for PAI-RBP1 also in AML.

Finally, bioinformatic integration of the datasets offered a way to meet the lack of direct overlap between molecules and genes which has been a common criticism of medium- and high-throughput screening methods. This revealed that MAPKAPK2, ACTB, HSP90AA1 and HSP90AB1 are evolutionary conserved hubs that allow cells to continue proliferation in the presence of VPA. Interruption of these hubs using small molecule inhibitors increased the effect of VPA in the human AML cell lines (Figure 3). Hence, these survival pathways should be further explored for development of new low toxicity therapeutic combinations with VPA.

## Supporting Information

Figure S1 **Pharmacokinetics of VPA in BN rats.** Serum levels were measured in **A**) the QD (once daily) high dose model (400 mg/kg) and **B**) the b.i.d. low dose model (170 mg/kg b.i.d.) at the indicated time points. Due to the administration regime of the drugs, the QD model (A) is represented by a steadily decaying curve, whilst the twice daily model (B) is represented by a biphasic curve with maximum serum concentration at one hour post treatment. Steady state VPA serum concentrations were calculated based on 4 and 5 times half-life of the drug giving 174–361 µM and 250–500 µM for the high and low dose respectively. Error bars represent standard errors of mean (SEM).
(PDF)

Figure S2 **Network of enriched biological processes identified by proteomics.** Phosphoproteins responding to VPA (*red*) as well as direct protein-protein interactors and indirect interactors (*black*) mediated via one neighbor were extracted using FunCoup and imported into Cytoscape in order to find enriched Biological Processes using the plug-in program BiNGO. The enriched processes were manually grouped into five major clusters to illustrate that “Regulation of translation and signaling”, “Regulation of ubiquitin ligase activity”, “APC-dependent proteasomal ubiquitin-dependent protein degradation”, “Purine nucleotide salvage” and “Energy and oxidative phosphorylation” are modulated by VPA in leukemic BN rats. The scale depicts color representation of significance by Benjamini-Hochberg correction, where white nodes are not significant, yellow *p*<0.05 and orange *p*<7×10^−7^.
(PDF)

Figure S3 **VPA combined with inhibition of conserved pathways results in synergistic cell death.** The AML cell lines MV4-11 and NB4 were treated with 1 mM VPA and **A**) inhibitor of HSP90 (17-DMAG, 5 nM), **B**) tubulin (vincristine, 0.5 µM) or **C**) actin polymerization (cytochalasin B, 2 µM) for 48 hours prior to analysis for cell death by the Annexin-V/Propidium Iodide viability assay. All combinations showed resulted in statistically significant synergism of drug interaction. Data are shown normalized to untreated control cells. Two-way ANOVA; * *p*<0.05, ** *p*<0.001. Error bars represent standard errors of mean (SEM).
(PDF)

Figure S4 ***C. elegans*** **mutant strains of VPA sensitizer genes are less sensitive to VPA.** *C. elegans* strains of genes mutant for VPA synthetic lethal and VPA sensitizer genes were obtained and treated with 0, 1 or 5 mM VPA for 72 hours. The mutants for synthetic lethal genes all showed decreased adult worm survival with increased concentrations of VPA. Sensitizer mutant strains showed no or little decrease in survival by 1 mM VPA, and increase in death only when treated with 5 mM VPA. However, this survival was substantially higher in sensitizer mutants (mean survival 46–60%) compared to synthetic lethal mutants (1–20% mean survival). Error bars represent SEM.
(PDF)

Figure S5 **VPA treatment induced global demethylation in 100-cell stage** ***C. elegans*** **embryos.** *C. elegans* strain AZ212, expressing GFP in fusion with H2B, was fed the empty vector L4440 (RNAi) and exposed to 15 mM VPA at L4 larval stage for 24 hours at 20°C. A) H4K8Ac appears at about the 100 cell stage in VPA-treated embryos but first at the 200 cell stage in untreated embryos. B) The embryos were fixed with acetone and methanol prior to staining with di-Methyl-Histone H3 (Lys36) antibody. At the 100-cell stage, baseline levels of methylation were seen in untreated worms. Global demethylation was observed after treatment with VPA suggesting a functional relationship between protein acetylation and lysine-specific methylation. Scale bar = 10 µM.
(PDF)

Figure S6 **VPA regulation of epigenetic marks.** AML cell lines were treated with 1 mM VPA for 48 hours and analyzed for EZH2, H3K27me3 and H2BK120ac expression. The mean intensity on one representative Western blot was calculated and normalized to beta-Actin. The numbers shown are in arbitrary units compared to MV4-11 control. **A**) The effect on VPA on EZH2 expression is not dependent on the expression of UTX. **B**) VPA increases the degree of H3K27 trimethylation in all cell lines except the UTX-null THP-1. **C**) VPA increases the degree of H2BK120 acetylation in all cell lines except THP-1 and MV4-11, where the level of acetylation is decreased.
(PDF)

Table S1 ***In silico*** **human AML gene expression screen investigated for effects on synthetic lethality by RNAi and VPA treatment in** ***C. elegans*** **.**
(DOC)

Table S2 **UTX-binding genes from the gene expression,** ***C. elegans*** **and BNML screen.**
(DOC)
