# Supplementary figures and images for: Cross-Species Functional Genomic Analysis Identifies Resistance Genes of the Histone Deacetylase Inhibitor Valproic Acid
Source: PLoS One. 2012 Nov 14;7(11):e48992. doi: 10.1371/journal.pone.0048992 (PMC3498369; doi:10.1371/journal.pone.0048992)

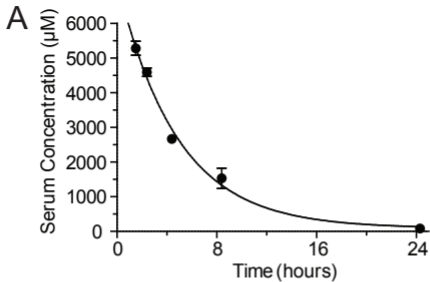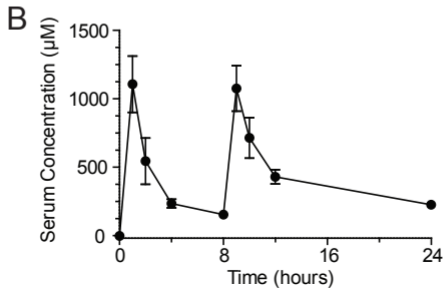

Supplement: Figure S1 — Pharmacokinetics of VPA in BN rats. Serum levels were measured in A) the QD (once daily) high dose model (400 mg/kg) and B) the b.i.d. low dose model (170 mg/kg b.i.d.) at the indicated time points. Due to the administration regime of the drugs, the QD model (A) is represented by a steadily decaying curve, whilst the twice daily model (B) is represented by a biphasic curve with maximum serum concentration at one hour post treatment. Steady state VPA serum concentrations were calculated based on 4 and 5 times half-life of the drug giving 174–361 µM and 250–500 µM for the high and low dose respectively. Error bars represent standard errors of mean (SEM). (PDF) [file pone.0048992.s001.pdf]

Figure S3

A

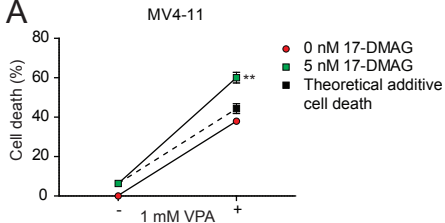

B

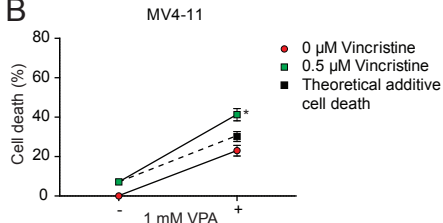

C

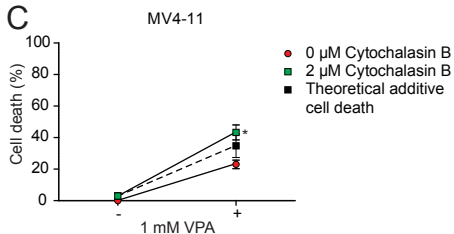

D

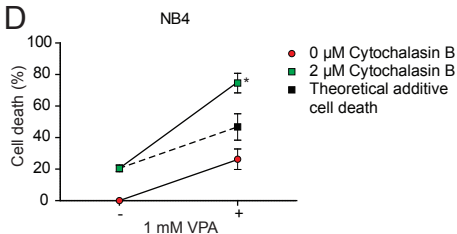

Supplement: Figure S3 — VPA combined with inhibition of conserved pathways results in synergistic cell death. The AML cell lines MV4-11 and NB4 were treated with 1 mM VPA and A) inhibitor of HSP90 (17-DMAG, 5 nM), B) tubulin (vincristine, 0.5 µM) or C) actin polymerization (cytochalasin B, 2 µM) for 48 hours prior to analysis for cell death by the Annexin-V/Propidium Iodide viability assay. All combinations showed resulted in statistically significant synergism of drug interaction. Data are shown normalized to untreated control cells. Two-way ANOVA; * p<0.05, ** p<0.001. Error bars represent standard errors of mean (SEM). (PDF) [file pone.0048992.s003.pdf]

Figure S4

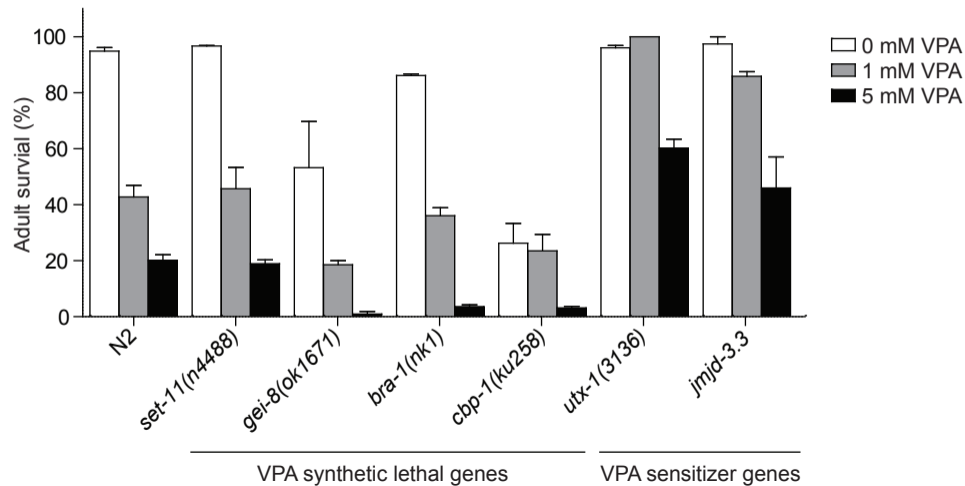

Supplement: Figure S4 — C. elegans mutant strains of VPA sensitizer genes are less sensitive to VPA. C. elegans strains of genes mutant for VPA synthetic lethal and VPA sensitizer genes were obtained and treated with 0, 1 or 5 mM VPA for 72 hours. The mutants for synthetic lethal genes all showed decreased adult worm survival with increased concentrations of VPA. Sensitizer mutant strains showed no or little decrease in survival by 1 mM VPA, and increase in death only when treated with 5 mM VPA. However, this survival was substantially higher in sensitizer mutants (mean survival 46–60%) compared to synthetic lethal mutants (1–20% mean survival). Error bars represent SEM. (PDF) [file pone.0048992.s004.pdf]

Figure S5

A

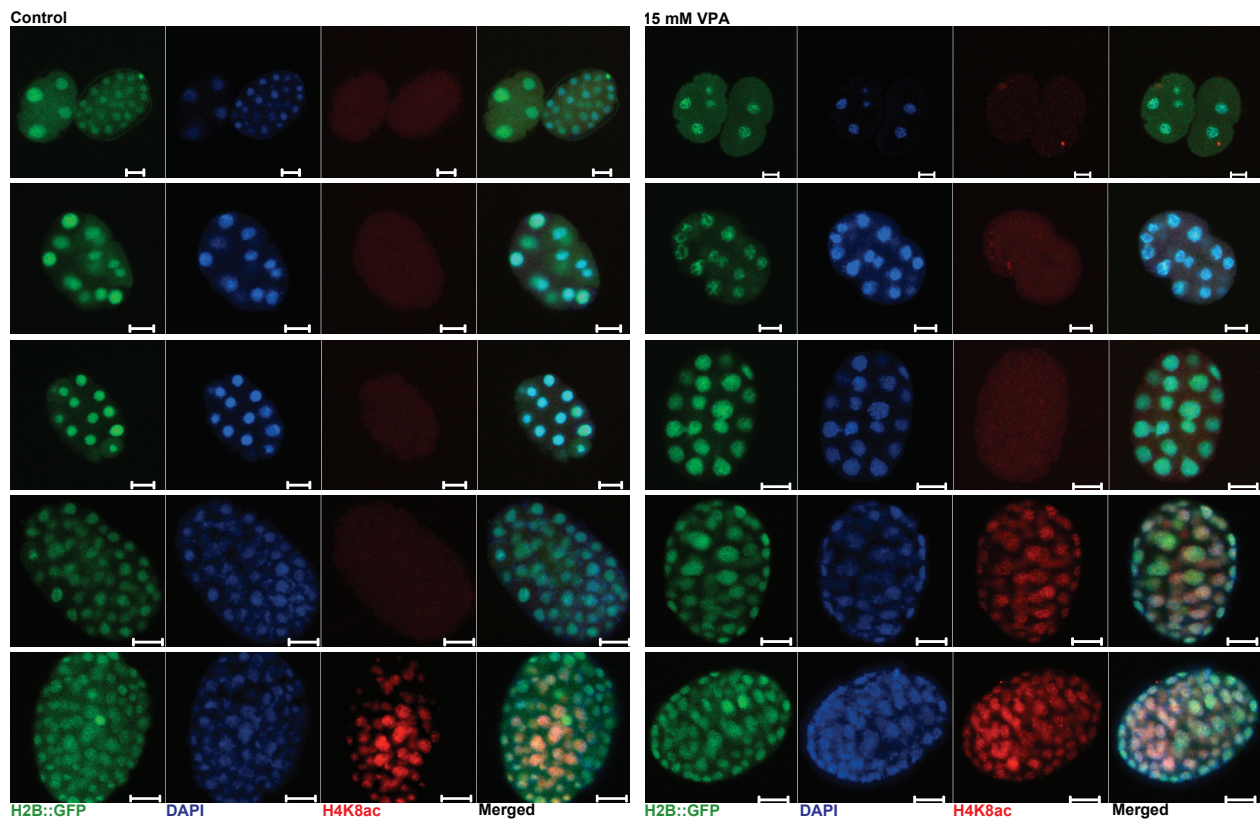

B

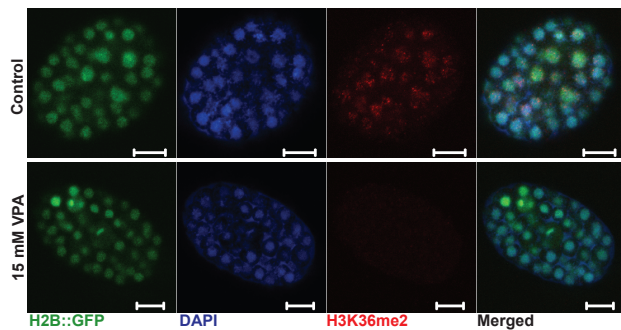

Supplement: Figure S5 — VPA treatment induced global demethylation in 100-cell stage C. elegans embryos. C. elegans strain AZ212, expressing GFP in fusion with H2B, was fed the empty vector L4440 (RNAi) and exposed to 15 mM VPA at L4 larval stage for 24 hours at 20°C. A) H4K8Ac appears at about the 100 cell stage in VPA-treated embryos but first at the 200 cell stage in untreated embryos. B) The embryos were fixed with acetone and methanol prior to staining with di-Methyl-Histone H3 (Lys36) antibody. At the 100-cell stage, baseline levels of methylation were seen in untreated worms. Global demethylation was observed after treatment with VPA suggesting a functional relationship between protein acetylation and lysine-specific methylation. Scale bar = 10 µM. (PDF) [file pone.0048992.s005.pdf]

Figure S6

A

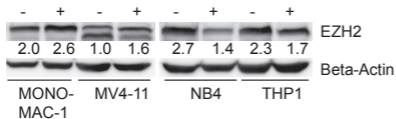

B

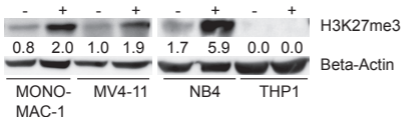

C

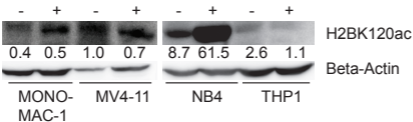

Supplement: Figure S6 — VPA regulation of epigenetic marks. AML cell lines were treated with 1 mM VPA for 48 hours and analyzed for EZH2, H3K27me3 and H2BK120ac expression. The mean intensity on one representative Western blot was calculated and normalized to beta-Actin. The numbers shown are in arbitrary units compared to MV4-11 control. A) The effect on VPA on EZH2 expression is not dependent on the expression of UTX. B) VPA increases the degree of H3K27 trimethylation in all cell lines except the UTX-null THP-1. C) VPA increases the degree of H2BK120 acetylation in all cell lines except THP-1 and MV4-11, where the level of acetylation is decreased. (PDF) [file pone.0048992.s006.pdf]
