# Supplementary material for: Cross-Species Functional Genomic Analysis Identifies Resistance Genes of the Histone Deacetylase Inhibitor Valproic Acid
Source: PLoS One. 2012 Nov 14;7(11):e48992. doi: 10.1371/journal.pone.0048992 (PMC3498369; doi:10.1371/journal.pone.0048992)
Supplement: Figure S2 — Network of enriched biological processes identified by proteomics. Phosphoproteins responding to VPA (red) as well as direct protein-protein interactors and indirect interactors (black) mediated via one neighbor were extracted using FunCoup and imported into Cytoscape in order to find enriched Biological Processes using the plug-in program BiNGO. The enriched processes were manually grouped into five major clusters to illustrate that “Regulation of translation and signaling”, “Regulation of ubiquitin ligase activity”, “APC-dependent proteasomal ubiquitin-dependent protein degradation”, “Purine nucleotide salvage” and “Energy and oxidative phosphorylation” are modulated by VPA in leukemic BN rats. The scale depicts color representation of significance by Benjamini-Hochberg correction, where white nodes are not significant, yellow p<0.05 and orange p<7×10^−7^. (PDF) [file pone.0048992.s002.pdf]

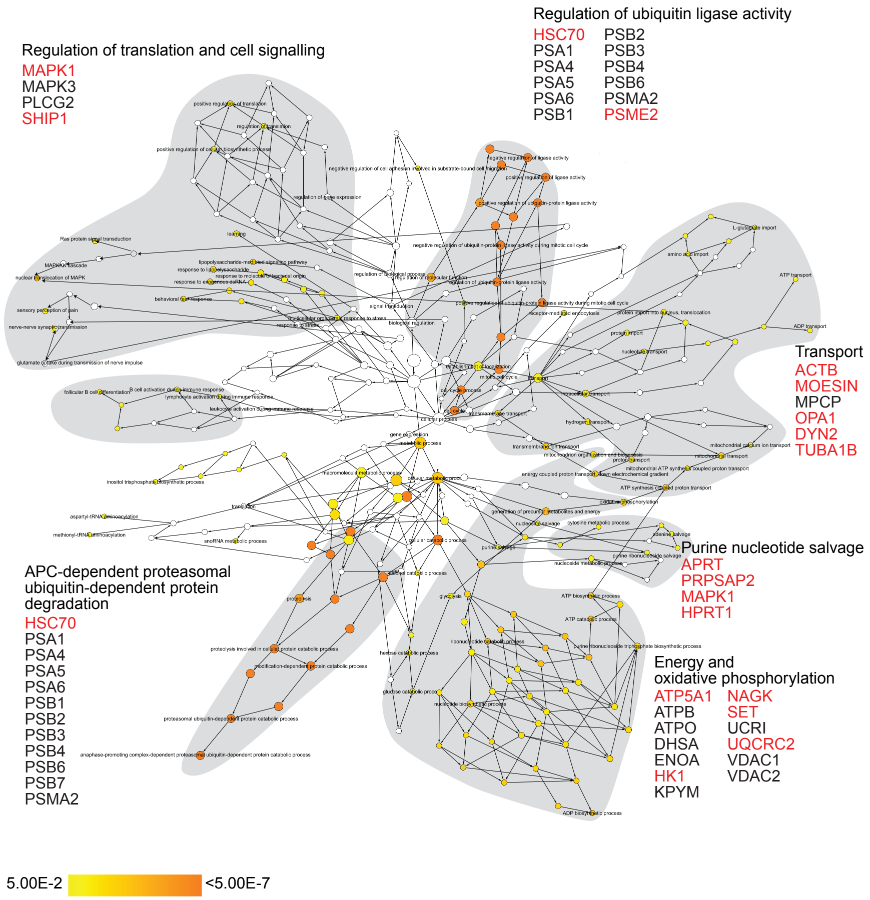
Regulation of ubiquitin ligase activity
HSC70
PSA1
PSA4
PSA5
PSA6
PSB1
PSB2
PSB3
PSB4
PSB6
PSMA2
PSME2
Regulation of translation and cell signalling
MAPK1
MAPK3
PLCG2
SHIP1
Transport
ACTB
MOESIN
MPCP
OPA1
DYN2
TUBA1B
Purine nucleotide salvage
APRT
PRPSAP2
MAPK1
HPRT1
APC-dependent proteasomal ubiquitin-dependent protein degradation
HSC70
PSA1
PSA4
PSA5
PSA6
PSB1
PSB2
PSB3
PSB4
PSB6
PSB7
PSMA2
Energy and oxidative phosphorylation
ATP5A1
ATPB
ATPO
DHSA
ENOA
HK1
KPYM
NAGK
SET
UCRI
UQCRC2
VDAC1
VDAC2
positive regulation of translation
regulation of translation
positive regulation of cellular biosynthetic process
regulation of gene expression
negative regulation of cell adhesion involved in substrate-bound cell migration
negative regulation of ligase activity
positive regulation of ligase activity
positive regulation of ubiquitin-protein ligase activity
negative regulation of ubiquitin-protein ligase activity during mitotic cell cycle
regulation of ubiquitin-protein ligase activity
positive regulation of ubiquitin-protein ligase activity during mitotic cell cycle
Ras protein signal transduction
learning
MAPKKK cascade
nuclear translocation of MAPK
lipopolysaccharide-mediated signaling pathway
response to lipopolysaccharide
response to molecule of bacterial origin
response to exogenous dsRNA
behavioral fear response
sensory perception of pain
nerve-nerve synaptic transmission
glutamate uptake during transmission of nerve impulse
regulation of biological process
regulation of molecular function
signal transduction
multicellular organismal response to stress
response to stress
biological regulation
receptor-mediated endocytosis
L-glutamate import
amino acid import
ATP transport
ADP transport
protein import into nucleus, translocation
protein import
nucleotide transport
establishment of localization
mitotic cell cycle
transport
intracellular transport
cell cycle process
cell cycle
cellular process
transmembrane transport
hydrogen transport
transmembrane ion transport
mitochondrion organization and biogenesis
proton transport
mitochondrial calcium ion transport
mitochondrial transport
mitochondrial ATP synthesis coupled proton transport
energy coupled proton transport, down electrochemical gradient
ATP synthesis coupled proton transport
oxidative phosphorylation
generation of precursor metabolites and energy
follicular B cell differentiation
B cell activation during immune response
lymphocyte activation during immune response
leukocyte activation during immune response
gene expression
metabolic process
inositol trisphosphate biosynthetic process
macromolecule metabolic process
cellular metabolic process
translation
aspartyl-tRNA aminoacylation
methionyl-tRNA aminoacylation
snoRNA metabolic process
nucleotide salvage
cytosine metabolic process
adenine salvage
purine salvage
purine ribonucleoside salvage
nucleoside metabolic process
cellular catabolic process
alcohol catabolic process
proteolysis
glycolysis
ATP biosynthetic process
ATP catabolic process
ribonucleotide catabolic process
purine ribonucleoside triphosphate biosynthetic process
hexose catabolic process
glucose catabolic process
nucleotide biosynthetic process
ADP biosynthetic process
proteolysis involved in cellular protein catabolic process
modification-dependent protein catabolic process
proteasomal ubiquitin-dependent protein catabolic process
anaphase-promoting complex-dependent proteasomal ubiquitin-dependent protein catabolic process
5.00E-2
<5.00E-7
