# Supplementary material for: Cross-Species Functional Genomic Analysis Identifies Resistance Genes of the Histone Deacetylase Inhibitor Valproic Acid
Source: PLoS One. 2012 Nov 14;7(11):e48992. doi: 10.1371/journal.pone.0048992 (PMC3498369; doi:10.1371/journal.pone.0048992)
Supplement: Table S1 — In silico human AML gene expression screen investigated for effects on synthetic lethality by RNAi and VPA treatment in C. elegans . (DOC) [file pone.0048992.s007.doc]

**Table S1. *In silico* human AML gene expression screen investigated for effects on synthetic lethality by RNAi and VPA treatment in *C. elegans***

| **Human gene** | **Gene array classification** | **Gene expression fold change** | ***C. elegans* gene** | **Synthetic lethal*** |
| --- | --- | --- | --- | --- |
| *ABCA5* | Non-responsive | *4.61* | *abt-4* | Y |
| *AGPAT4* | Non-responsive | *4.192* | *acl-8* | Y |
|  |  |  | *acl-10* | Y |
| *BAG2* | Non-responsive | *3.096* | *unc-23* | Y |
| *COCH* | Non-responsive | *3.879* | *cut-6* | Y |
| *FLIPT1* | Non-responsive | *6.11* | *oct-2* | Y |
|  |  |  | *K05F1.6* | Y |
| *WDR35* | Non-responsive | *3,959* | *ifta-1* | Y |
| *EID3* | Responsive | *3.803* | *H21P03.2* | Y |
| *KCNA3* | Responsive | *5.753* | *shk-1* | Y |
| *MAD1 ortholog* | Responsive | *3.229* | *mdf-1* | Y |
| *SERPINF1* | Responsive | *4.09* | *srp-6* | Y |
| *SMAD3* | Responsive | *2.572* | *sma-3* | Y |
| *AKT3* | Responsive | *6.399* | *akt-1* | - |
| *IL12RB2* | Responsive | *6.026* | *let-805* | - |
| *NDRG2* | Responsive | *6.245* | *ZK1073.1* | - |
| *SERPINF1* | Responsive | *4.09* | *srp-1* | - |
|  |  |  | *srp-2* | - |
| *SMAD3* | Responsive | *2.572* | *daf-3* | - |
|  |  |  | *daf-8* | - |
|  |  |  | *sma-4* | - |
|  |  |  | *sma-2* | - |
|  |  |  | *daf-14* | - |
| Control |  |  | L4440 | - |

*Synthetic lethality of *C. elegans* treated with RNAi in combination with 15 mM VPA is indicated by Y.
