# Supplementary material for: Cross-Species Functional Genomic Analysis Identifies Resistance Genes of the Histone Deacetylase Inhibitor Valproic Acid
Source: PLoS One. 2012 Nov 14;7(11):e48992. doi: 10.1371/journal.pone.0048992 (PMC3498369; doi:10.1371/journal.pone.0048992)
Supplement: Table S2 — UTX-binding genes from the gene expression, C. elegans and BNML screen. (DOC) [file pone.0048992.s008.doc]

**Table S2. UTX-binding genes from the gene expression, *C. elegans*** and BNML screen*.

| **Gene** | **Source** |
| --- | --- |
| ACBA5 | Gene expression analysis |
| EID3 | Gene expression analysis |
| IL12RB2 | Gene expression analysis |
| WDR35 | Gene expression analysis |
| ASPH | Unpublished results, gene expression analysis |
| CKS2 | Unpublished results, gene expression analysis |
| ERMAP | Unpublished results, gene expression analysis |
| NCBP1 | Unpublished results, gene expression analysis |
| PPP4R1 | Unpublished results, gene expression analysis |
| TMEM34 | Unpublished results, gene expression analysis |
| WDR3 | Unpublished results, gene expression analysis |
| C21orf66 | *C. elegans* RNAi screen |
| H3F3B | *C. elegans* RNAi screen |
| MYST3 | *C. elegans* RNAi screen |
| NCOR1 | *C. elegans* RNAi screen |
| SMARCA1 | *C. elegans* RNAi screen |
| ATP5A1 | BNML phosphoprotein screen |
| PRPSAP2 | BNML phosphoprotein screen |

* Genes overlapping with UTX-binding genes from Wang *et al*.

**References**

1. Stapnes C, Ryningen A, Hatfield K, Oyan AM, Eide GE, et al. (2007) Functional characteristics and gene expression profiles of primary acute myeloid leukaemia cells identify patient subgroups that differ in susceptibility to histone deacetylase inhibitors. Int J Oncol 31: 1529-1538.

2. Wang JK, Tsai MC, Poulin G, Adler AS, Chen S, et al. (2010) The histone demethylase UTX enables RB-dependent cell fate control. Genes Dev 24: 327-332.
